# Multi-Pharmaceutical Activities of Chinese Herbal Polysaccharides in the Treatment of Pulmonary Fibrosis: Concept and Future Prospects

**DOI:** 10.3389/fphar.2021.707491

**Published:** 2021-08-17

**Authors:** Xianbo Wu, Jianli Huang, Jie Wang, Yihua Xu, Xinwei Yang, Minghan Sun, Jianyou Shi

**Affiliations:** ^1^ School of Sports Medicine and Health, Chegdu Sport University, Chengdu, China; ^2^ Guizhou University of Traditional Chinese Medicine, Guiyang, China; ^3^ Chengdu University of Traditional Chinese Medicine, Chengdu, China; ^4^ Central of Reproductive Medicine, Department of Obstetrics and Gynecology, School of Medicine, Sichuan Academy of Medical Sciences and Sichuan Provincial People’s Hospital, University of Electronic Science and Technology of China, Chengdu, China; ^5^ Personalized Drug Therapy Key Laboratory of Sichuan Province, Sichuan Academy of Medical Science and Sichuan Provincial People’s Hospital, School of Medicine of University of Electronic Science and Technology of China, Chengdu, China

**Keywords:** pulmonary fibrosis, traditional Chinese medicine, polysaccharide, transforming growth factor-β, extracellular matrix, collagen-1, biological activity

## Abstract

Pulmonary fibrosis is a fatal chronic progressive respiratory disease, characterized by continuous scarring of the lung parenchyma, leading to respiratory failure and death. The incidence of PF has increased over time. There are drugs, yet, there are some limitations. Hence, it is of importance to find new therapies and new drugs to replace the treatment of pulmonary fibrosis. In recent years, there have been a great number of research reports on the treatment of traditional Chinese medicine polysaccharides in various system fields. Among them, the treatment of PF has also gained extensive attention. This review summarized the source of polysaccharides, the drug activity of traditional Chinese medicine, and the protective effects on targets of Pulmonary fibrosis. We hope it can inspire researchers to design and develop polysaccharides, serving as a reference for potential clinical therapeutic drugs.

## Introduction

**FIGURE 1:**
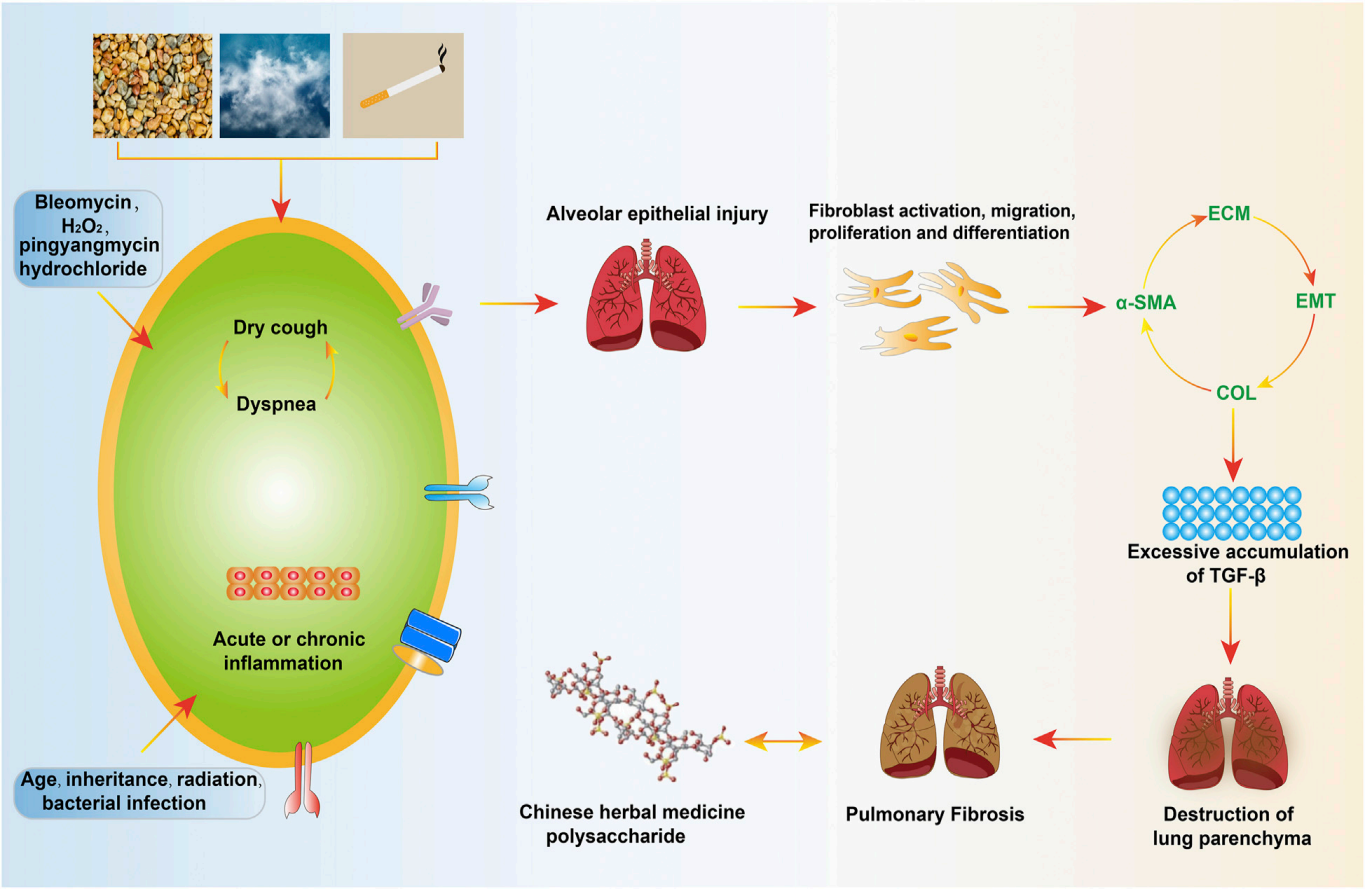
Pathogenic factors and development process of pulmonary fibrosis.

Pulmonary fibrosis (PF) is a large category of pulmonary diseases refers to the proliferation of fibroblasts and the accumulation of a large amount of extracellular matrix (ECM), accompanied by inflammatory damage, and tissue structure destruction, which can cause breathing difficulties, cough, hypoxemia, and hinder gas exchange, eventually leading to respiratory failure (Phan et al., 2021). PF’s pathogenesis has undergone fibroblast activation, migration, proliferation and differentiation into myofibroblasts, inducing ECM aggregation, destroying the pulmonary parenchyma, changing the expansion of the pulmonary and the diffusion of O2/CO2, resulting in respiratory abnormalities caused by insufficient gas exchange (limitation reduced pulmonary volume and diffusing capacity) (Ruigrok et al., 2021). The triggering factors for fibrosis include genetic susceptibility and other risk factors, such as persistent viruses, bacterial infections, cigarettes, drug damage, and other medical diseases. Hereditary factors account for the largest proportion among them, one that the cause is unknown and the most common type of the disease is called idiopathic PF. (Richeldi et al., 2017; Majewski and Piotrowski, 2020). Radiology of chest high-resolution CT showed that about 90% of patients had interstitial changes, the histology of pulmonary biopsy showed that scar tissue replaced the normal pulmonary parenchyma, which was also used as a standard for the diagnosis of PF (Solomon et al., 2013). The occurrence and development of PF and the decline of pulmonary function are closely related to the survival rate. According to reports, the incidence of PF is increasing year by year and is positively correlated with age, with males higher than females (Hutchinson et al., 2015). With ageing, patients will inevitably experience the gradual loss of physiological integrity, the decrease of steady-state controlling ability and the increase of vulnerability to death. If not taking corresponding treatment measures after diagnosis, most patients’ pulmonary function, if not all, will be progressively and irreversibly worsening, and their survival time is within 3–5 years (Lederer and Martinez, 2018) **(** Figure 1 **)**.

Currently, only two anti-fibrosis drugs, named pirfenidone and nintedanib, have been approved by the US Food and Drug Administration to treat PF, but, due to their side effects (headache, nausea, diarrhea, skin rash, and impaired liver function) and high cost, they cannot meet the medical needs (Galli et al., 2017). Lung transplantation can improve the survival rate, but the limited organ supply and the complexity of the surgery and medical treatment make it affordable for only a small number of patients (Ryu et al., 2014). Colchicine, prednisone, and cyclophosphamide were also used to reduce the incidence and mortality of PF, studies on PF reported (Miniati and Matucci Cerinic, 2007; Fiorucci et al., 2008). In the case of acute exacerbations, international guidelines recommend high-dose glucocorticoids, but there are no data to prove its safety and effectiveness (Song et al., 2011). It has also been suggested that the therapeutic effects of prednisolone, cyclophosphamide, interferon-1b, and N-acetylcysteine have no obvious advantages, but various adverse reactions and increase the economic burden of patients (Datta et al., 2011; Canestaro et al., 2016; Raimundo et al., 2016). Therefore, it is critical to developing new therapies and new drugs for PF. Many traditional Chinese medicines (TCM) have played a certain role in the treatment of various systems of diseases (Huang et al., 2018). Chinese herbal extracts also have many effective pharmacological ingredients to treat PF (Ji et al., 2016; Huo et al., 2020). In the past 30 years, the pharmacological activity of polysaccharides has attracted increasing attention (Layek and Mandal, 2020). Japanese scholar, Chihara Hiroro, firstly discovered that lentinan has an anti-tumor effect in 1968, thus striking the research boom of natural active polysaccharides (Chihara et al., 1969). Polysaccharides are natural macromolecular compounds composed of monosaccharides. They, with abundant biological activities and obviously low toxicity, are one of the main active components of Chinese medicine. Polysaccharides have received wide attention thanks to their effective biological activities and multiple molecular targets (Muhamad et al., 2019; Cao et al., 2020; Mohammed et al., 2021). Studies have shown that polysaccharides have a variety of biological activities, such as anti-tumor, anti-oxidation, anti-radiation, anti-virus, and hypoglycemic effects (Mu et al., 2021; Niu et al., 2021). The development and application of polysaccharides in Chinese herbal medicine have enriched the treatment methods of modern medicine. Nowadays, TCM polysaccharides have been widely used clinically to treat related diseases, such as astragalus polysaccharide injection, ganoderma polysaccharide injection, ginseng polysaccharide injection, etc. (Li et al., 2019b). As expected, TCM polysaccharides have protective effects on PF. For example, astragalus polysaccharides (APS) can reduce the degree of PF by inhibiting transforming growth factor-β1 (TGF-β1) *in vitro* and *in vivo* (Zhang, Xu, 2020). Ganoderma lucidum polysaccharides (GIP) inhibit bleomycin (BLM)-induced adult male SD rats by improving lung antioxidant capacity (Chen et al., 2016). Therefore, Chinese herbal polysaccharides provide a new way to discover and develop anti-PF.

**FIGURE 2:**
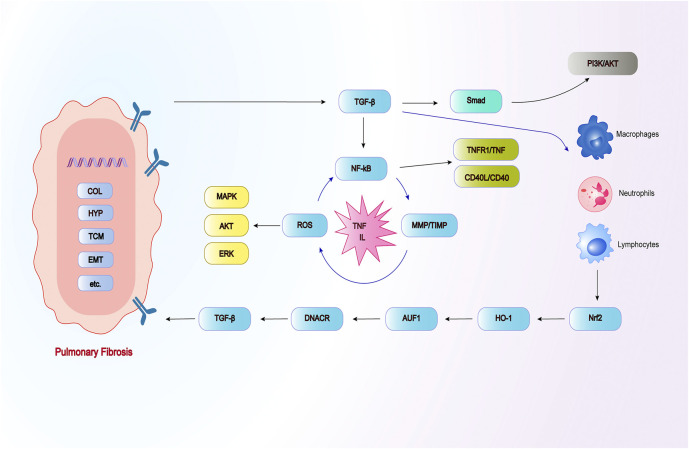
The occurrence and development of PF can be related to these pathways.

Repeated local micro-injuries play a fatal role in the aging alveolar epithelium, these micro-injuries start the connection of abnormal epithelial cell with fibroblast, induce myofibroblasts that produce matrix, and remodel a large amount of ECM accumulation and lung interstitial. There are many sources of myofibroblasts, including resident mesenchymal cell proliferatsion, pulmonary interstitial cells, circulating fibroblasts, epithelial-mesenchymal transition (EMT) and endothelial-mesenchymal transition (Todd et al., 2012). The histological characteristics of PF are excessive deposition of matrix collagen, increased fibrosis area and increased hydroxyproline (HYP) content, which can damage the normal structure and function of the lung. Activated alveolar epithelial cells secrete large amounts of fibrogenic growth factors and cytokines, including TGF-β1 and platelet-derived growth factors. TGF-β1 is an important regulator of fibrogenesis, which can activate fibroblasts and transform them into myofibroblasts (Organ et al., 2015), the initiation and regression of myofibroblasts herald disease progression. TGF-β1 not only promotes ECM deposition, but also concentrates it in the accumulated matrix, thereby accelerating it the pro-fibrotic reaction. The current pathogenesis of PF presumably includes abnormal accumulation of TGF-β, cell recruitment, apoptosis, inflammatory factors, oxidative stress, and the imbalance of matrix metalloproteinase/tissue inhibitor of metalloproteinase (MMP/TIMP) (Ryu et al., 2014; Spagnolo et al., 2021) (Figure 2). Therefore, the use of various activities of Chinese herbal polysaccharides brings hope to the treatment of PF through these targets. Chinese herbal polysaccharides can participate in reversing the down-regulation of TGF-β and protecting PF, and have broad prospects in the field of anti-PF research.

### Polysaccharides

#### Ginseng Polysaccharide (GPS)

**FIGURE 3:**
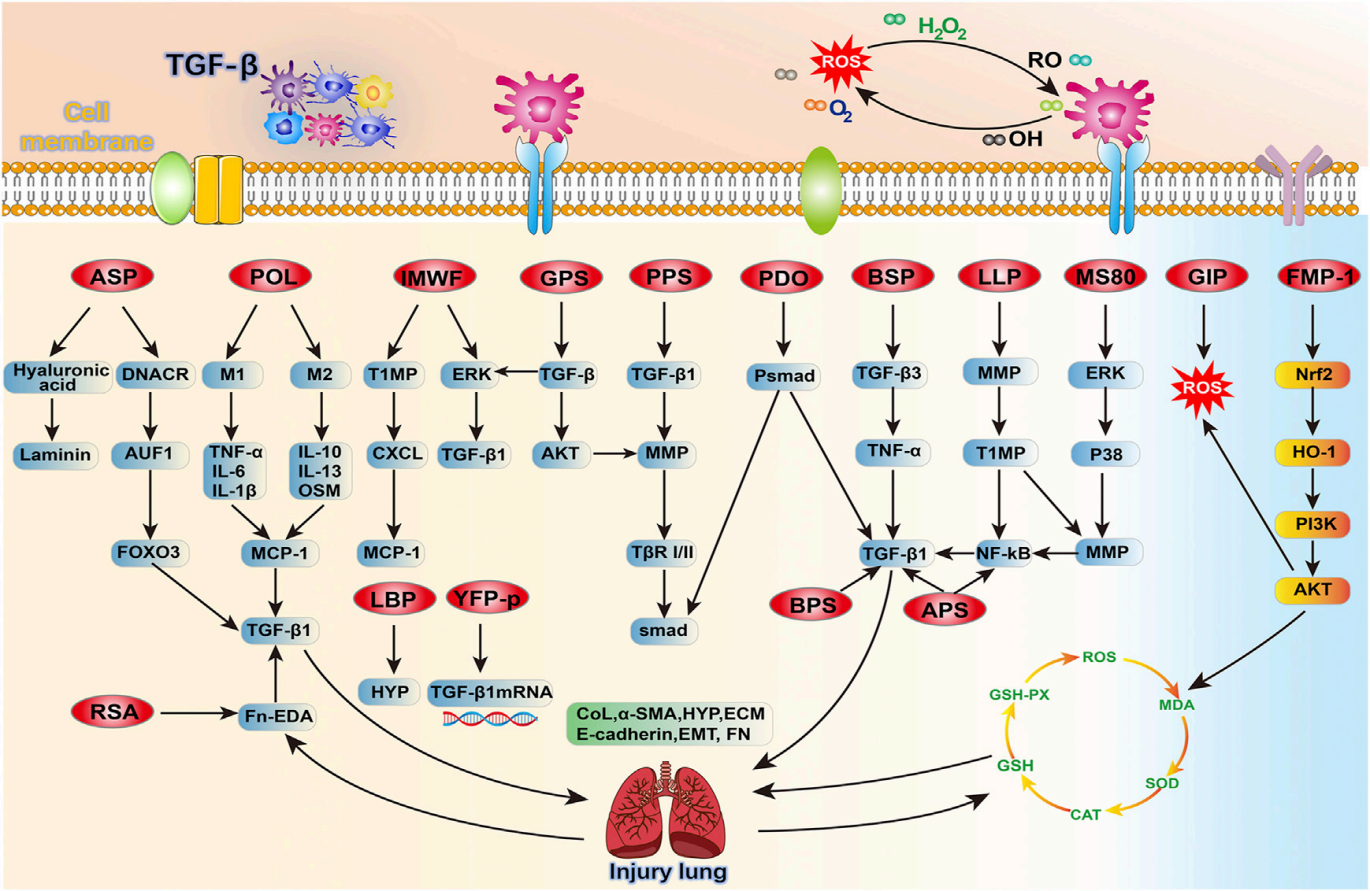
An overview of the target signaling pathways of 16 Chinese herbal polysaccharides that antagonize PF, mainly by reversing TGF-β and improving ROS to treat pulmonary fibrosis, and further linking the relationship with these related pathways.

GPS is a polysaccharide isolated from ginseng roots, with an average molecular weight (Mw) of 1.5 × 10^6^ Da, mainly composed of Glc and Gal (over 90%, w/w) and 5–8% Man and Ara (Chen et al., 2020; Ahn et al., 2011; Ahn et al., 2011; Chen et al., 2020). It makes a variety of immunomodulatory functions, such as anti-oxidation, anti-tumor, anti-cancer and anti-adhesion (Akhter et al., 2018; Xiong et al., 2019). The Smad pathway is essential for TGF-β mediated signal transduction (Wilkes et al., 2005). Studies have shown that GPS inhibits the phosphorylation of Smad2 and Smad3 in fibroblasts through TGF-β, but has no response to the levels of Smad6 and Smad7, weakens the phosphorylation level of extracellular signal-regulated kinase/protein kinase B (ERK/AKT) and reverses the synthesis of collagen (COL)-1 and fibronectin (FN) also significantly reduces the protein expression of TGF-β1 receptor (TβRI) and TβRII in NIH/3T3 cells, preventing TβRII from its known that the protein expression of receptor TβRIII decreases, and it also inhibits the expression of a-smooth muscle actin (a-SMA) in IMR-90 and WI-38 cells (Ahn et al., 2011). Later, studies reported that ginsenosides can reduce EMT of lung tissue by inhibiting TGF-β1/Smad pathway (Guan et al., 2017). There are further reports that ginsenosides can prevent renal fibrosis (Li et al, 2018b). Recent observers have shown that ginseng can prevent liver, lung, kidney and myocardial fibrosis through TGF-β (Liu et al., 2020a). Therefore, the possible mechanism of GPS anti-PF is that the downstream Smad2 and Smad3 signals of TGF-β1 and its TβRI and TβRII can achieve the treatment purpose, and reduce the phosphorylation of ERK and AKT, and reduce the level of MMPs, which is beneficial to lung tissue damage (Figure 3 and Table 1).

**TABLE 1:** Overview of the research progress of Chinese herbal medicine polysaccharides in the treatment of PF.

Name	Monosaccharides composition	Mw (kDa)	Molecular structure	Experimental model	Anti-PF effect	References
GPS	Glc and Gla (over 90%, w/w) and 5–8% Man and Ara	1.5 × 10^6^ Da	—	*In vivo*: BLM induced male C57BL/6 mice. *In vitro*: TGF-β1 induced NIH/3T3 cells and IMR-90 and WI-38 cells	Through the downstream Smad2 and Smad3 signals of TGF-β1 and its TβR I and TβRⅡ, the treatment is achieved, which the levels of COL and α-SMA decreased, weaken the phosphorylation of ERK and AKT, reduce the level of MMPs to reduce lung tissue damage	(Ahn et al., 2011; Akhter et al., 2018; Xiong et al., 2019; Chen et al., 2020)
BPS	Man, Rha, Glc, Fru, Ara	(8–10) × 10^4^ Da	—	*In vitro*: TGF-β induced A549 cells	BPS can reduce the inflammation of A549 cells by down-regulating TGF-β signaling to combat PF.it can reduce HYP and COL content, down-regulate the expression of a-SMA, and up-regulate the level of E-cadherin	(Lv et al., 2013; Yan et al., 2017; Feng et al., 2018; Zhan et al., 2020)
APS	Rha:Ara: Glc:Gal:GalA = 0.03:1.00:0.27:0.36:0.30	1,334 kDa	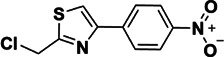 Chemical structure of APS: 2 -(Chloromethyl)-4-(4-nitrophenyl)-1,3-thiazole	*In vivo*: BLM induced male C57BL/6 mice. *In vitro*: TGF-β1 induced A549 cells	Regulate EMT and HYP content through the TGF-β1/NF-kB pathway, and reduce the area of a-SMA, COL deposition and fibrosis, and increase E-cadherin	(Bao et al., 2018; Ren et al., 2018; Jia et al., 2019; Liu et al., 2019a; Zhang et al., 2020)
PPS	Component consisting of (1 → 3)-β-glucan backbone and (1 → 6)-β-glucopyranose side chains	1.6 × 10^5^ Da	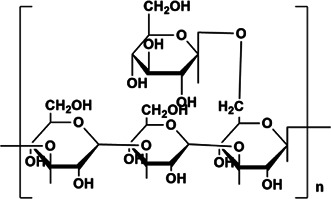 Chemical structure of PPS.	*In vivo*: BLM induced male C57BL/6 mice. *In vitro*: TGF-β1 induced in HLFs cells	The further deterioration of PF was prevented by TGF-β1/Smad2/3/TβRI and TβRII/MMP signaling, resulting in the decrease of α-SMA, MMP2/9, FN, COL-1/3 and ECM.	(Li et al., 2010; Zong et al., 2012; Guo et al., 2019; Jiang et al., 2020)
RSA	Ara:Rha:Xyl: Glc:Gal:GalA = 1.00:3.23:0.26:0.34:0.84:10.24	50,000 Da	—	*In vitro*: TGF-β1 induced A549 cells	RSA inhibits the process of TGF-β1 inducing A549 cells and down-regulates the expression of Fn-EDA.	(Han et al., 2002; Li et al., 2016; Yang et al., 2016; Song et al., 2019)
PDO	Man:Glc = 5.9:1, it is composed of (1→4)-linked Man and (1→4)-linked Glc	1.78 × 10^5^ Da	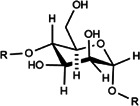 (1→4)-linked Man 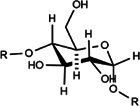 (1→4)-connected Glc	*In vivo*: BLM induced adult male Sprague Dawley rats. *In vitro*: TGF-β1 induced type II alveolar epithelial cells (RLE-6TN, CRL-2300)	PDO can reduce the number of neutrophils in rat lung tissue induced by BLM, improve inflammation and fibrosis, enhance Smad2/3 and inhibit the expression of pSmad2/3 protein through TGFβ1, thereby inhibiting the type II rat alveolar epithelial cells (RLE-6TN, CRL-2300) induced by TGF-β1 fibronectin and COL-1	(He et al., 2016; Wang et al., 2017a; Chen et al., 2018; Yu et al., 2018c; Liu et al., 2019b)
BSP	BSP-1:Man:Glc = 4.0:1.0, BSP-2: Man:Glc = 3.0:1.0, the backbone of BSP-1 and BSP-2 were consisted of β-1,4-linked D-Man residues and β-1,4-linked D-Glc residues	BSP: 2.23 × 10^5^ kDa, BSP-1:8.354 × 10^4^ Da, BSP-2:1.26 × 10^4^ Da	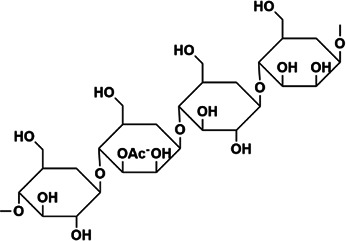 The main residues sequence of BSP-1 was deduced to be: →4)-β-D-Glcp (1→4)-β-D-Manp (1→4)-β-D-Manp (1→4)-β-D-Manp (1→4)-β-D-Manp (1→. The O-acetyl group position was also attached to C-3 of residue B (Man). 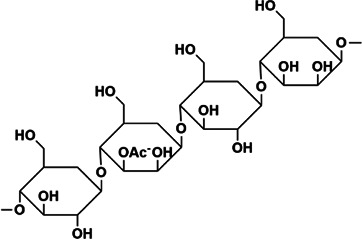 The residues sequence of BSP-2 was deduced to be: →4)-β-D-Glcp (1→4)-β-D-Manp (1→4)-β-D-Manp (1→4)-β-D-Manp (1→	*In vivo*: BLM induced male SD rats	BSP can reduce cell recruitment to protect lung tissue inflammation, HYP, a-SMA content is significantly reduced, and it also improves TGF-β3 and TNF-β1	(Guo et al., 2016; He et al., 2017; Wang et al., 2019b; Liu et al., 2020b)
MS80	New type of sulfated oligosaccharide (1→4α-d-Glc)	8,000 Da	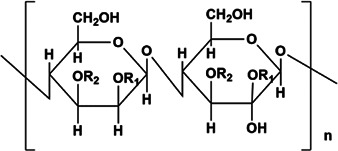 Chemical structure of MS80. R1 = SO3Na, R2 = SO3Na or H, n = 10∼15	*In vivo*: BLM induced pathogen-free adult Wistar rats. *In vitro*: TGF-β1 induced HEPF cells	MS80 can improve PF through TGF-β1/MMP/ERK/NF-kB/p38, prevent COL deposition, and reduce COL and EMT content	(Jiang and Guan, 2009; Zhou et al., 2016; Chen et al., 2020)
LMWF	Rha, Fuc, Xly, Man, Glc, Gal, GlcA, Gala, the linkage is backbone →3)-Galp-(1→, →6)-Glcp-(1→, →6)-Galp-(1→, →3,6)-manp-(1→ with →3)-Fucp-(1→, →4)-Glcp-(1→ and sulfated end units. Consists of (1→3)-linked α-thiopropionyl residues or alternating (13→)- and (→14)-linked α-thiophosphoryl residues	8–10 kDa	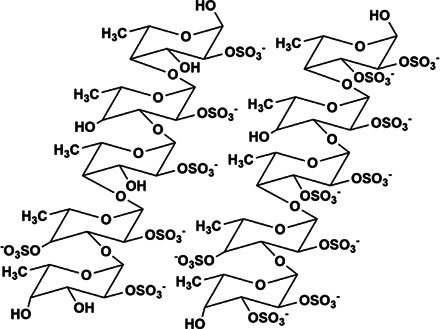 The overview of fucoidan regulation on signaling molecules	*In vivo*: BLM-induced male C57BL/6 mice. *In vitro*: TGF-β1 induced A549 cells	LMWF treats PF through TGF-β1/ERK signaling and improving inflammatory cytokines in pathological lung tissue, attenuates the expression of lung EMT phenotype a-SMA and HYP.	(Senthilkumar and Kim, 2014; Haddad et al., 2015; Wang et al., 2019a; Wang et al., 2018; Yu et al., 2018a; Zheng et al., 2018; Chen et al., 2020)
ASP	GalA:Glc:Ara:Gal = 1.00:1.70:1.85:5.02, its main chain is composed of (1→3)-connected Galp, (1→6)-connected Galp and 2-OMe-(1→6)-connected Galp, with three branches connected to 2-OMe-(- 1→6) link Galp and terminate with GlcpA and Araf	80 kDa, 85 kDa	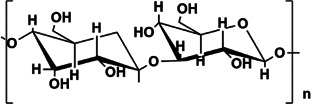 Chemically modified with branched LMW PEI (1,200 Da) to obtain cationic ASP.	*In vivo*: BLM induced healthy Wistar rats and Sprague-Dawley rats. *in vitro*: TGF-β1 induced alveolar type Ⅱ epithelial (RLE-6TN) cells	ASP can inhibit EMT and PF through the TGF-β1/DANCR/AUF-1/FOXO3 regulatory axis, reduce and reverse the expression levels of α-SMA, COL and E-cadherin	(Deng et al., 2013a; Zhang et al., 2016; Li et al., 2020a; Qian et al., 2020; Wang et al., 2010; Xu et al., 2021)
POL	Gal: Man:Glc = 5.30:13.38:81.31	3.2 × 10^5^ Da	—	*In vivo*: BLM induced male C57BL/6 mice	Improve lung inflammation by inhibiting the excessive recruitment of M1 and M2 under TGF-β1 to achieve a therapeutic effect, reducing the levels of MCP-1, TNF-α, IL-1β, IL-6, IL-10,IL-13, and OSM.	(Zhang et al., 2017; Ying-Mei et al., 2020; Zhou et al., 2020)
LBP	Glycan backbone is mainly represented by α-(1→4)-GalA, α-(1→6)-Glc, β-(1→3)-Galp (typical arabinogalactan protein) and β-(1→6)-Giap, other structures that are less representative are α-(1→5)-Ara and β-(1→4)-Galp	10–2,300 kDa	—	*In vivo*: BLM induced C57BL/6 mice	Inhibit the increase of HYP content in lung tissue when PF occurs, inhibit the increase of ECM, and reduce the expression levels of α-SMA and COL-1	(Liu et al., 2016b; Masci et al., 2018; Huang et al., 2019; Tian et al., 2019)
FMP-1	Man:Glc:Gal = 1.00:7.84:1.24, the main chain is 1,4-linked Glcp and 1,6-connected Galp composition	4.7 × 10^3^ Da	—	*In vitro*: H202 induced human alveolar epithelial cells (A549)	FMP-1 can be used as a natural potential antioxidant to treat PF through the Nrf2/HO-1/PI3K/AKT signaling pathway, which can weaken MDA and ROS levels, increase SOD enzyme activity and total antioxidant capacity	(Liu et al., 2016a; Li et al., 2017; Cai et al., 2018; Li et al., 2018c)
GIP	Gal:Rha:Glc = 1.00:1.15:3.22, backbone structure is mainly composed of 1,2-linked β-L Rhap, 1,3,6-linked aD-Galp, 1,2,6-linked aD-Glcp and 1-link aD-Glcp	78 kDa	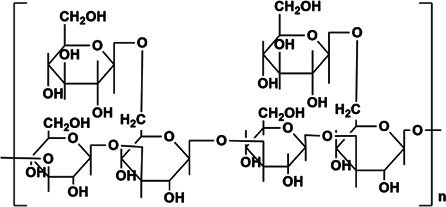 Structure of β-glucan in GIP.	*In vivo*: BLM induced adult male SD rats	Improve the status of PF by improving the antioxidant capacity of the lung, reducing inflammatory cell infiltration and collagen deposition, improving the content of CAT and SOD, increasing the levels of GSH, GSH-Px, and reducing the content of MDA and HYP in lung tissue	(Pan et al., 2012; Chen et al., 2016; Wang et al., 2017b; Sohretoglu and Huang, 2018; Zeng et al., 2018; Zeng et al., 2019)
LLP	LLP-1:Glc:Man = 1:2, LLP-2:Glc:Man = 1:1, LLP-3:Ara:Gal: Glc:Man = 2:2:2:1	LLP-1:2.25 × 10^6^ Da, LLP-2:2.02 × 10^6^ Da, LLP-3:2.08 × 10^6^ Da	—	*In vivo*: BLM induced SPF Kunming mice	Inhibit MMP-9 and TIMP-2 in lung tissue of BLM pulmonary fibrosis mice, reduce HYP content and COL level, and inhibit the expression of TNF-a, NF-kB in lung tissue of pulmonary fibrosis	(Chen et al., 2014; Hou et al., 2016; Li et al., 2020b; Luo et al., 2013a)
YPF-p	-	—	—	*In vivo*: pingyangmycin hydrochloride induced Wistar rats and BLM induced healthy adult female Sprague-Dawley rats	YPF-p treats PF by down-regulating the expression of TGF-β1 mRNA and improving the excessive aggregation of fibroblasts induced by TGF-β1, which reduces the expression of HYP, COL and a-SMA in lung tissue	(Wang et al., 2009; Xu et al., 2014; Sun et al., 2016; Fan et al., 2017)

### Basil Polysaccharide (BPS)

BPS is extracted from the Chinese herbal medicine basil, basil is a daily dish prepared in summer. BPS is a compound polysaccharide composed of fructose. It is mainly composed of Man, Rha, Glc, Fru, and Ara, the Mw is (8–10)×10^4^ Da (Zhan et al., 2020). It has anti-tumor, anti-oxidation, anti-inflammatory, hypolipidemic, anti-diabetic and anti-liver cancer effects (Lv et al., 2013; Feng et al., 2018). Proves have shown that BPS has a potential inhibitory effect on the PF of human A549 cells induced by TGF-β, and down-regulates the expression of a-SMA and COL-1, up-regulates E-cadherin levels, and reduces HYP content (Yan et al., 2017), and proposed that BPS has an inhibitory effect on EMT (Feng et al., 2019). Therefore, BPS can reduce the inflammation of A549 cells by down-regulating TGF-β signaling to combat PF.

### *Astragalus* polysaccharide (APS)

*Astragalus* as a medicinal and tonic food can keep fit and improve health. APS is a water-soluble polysaccharide isolated and purified from astragalus, the Mw is 1,334 kDa, composed of Rha, Ara, Glc, Gal, and GalA, the molar ratio is 0.03:1.00:0.27:0.36:0.30. The backbone of APS consisted of 1,2,4-linked Rhap, α-1, 4-linked Glcp, α-1, 4-linked GalAp6Me, β-1, 3, 6-linked Galp, with it branched at O-4 of the 1,2,4-linked Rhap and O-3 or O-4 of β-1, 3, 6-linked Galp. The side chains mainly consisted of α-T-Araf and α-1, 5-linked Araf with O-3 as branching points, having trace Glc and Gal, the terminal residues were T-linked Araf, T-linked Glcp, and T-linked Galp (Bao et al., 2018), its chemical structure is 2-(chloromethyl)-4-(4-nitrophenyl)-1,3-thiazole (Liu et al., 2019a). APS has been reported that have a variety of biological activities, including anti-inflammatory, anti-tumor, anti-diabetic and anti-oxidant (Ren et al., 2018; Jia et al., 2019). Previous reports suggested that astragalus injection and astragaloside IV have therapeutic effects on fibrosis (Qian et al., 2018). APS can protect renal fibrosis (Ren et al., 2019). *Astragalus* can improve EMT through β-catenin (Yu et al., 2018b). The overproduction of TGF-β1 is always closely related to PF, and TGF-β1 plays a key role in inducing EMT (Walton et al., 2017), nuclear factor-kB (NF-kB) regulates the expression of a variety of cytokines and inflammatory mediators, by inhibiting the expression of TGF-β1/NF-kB to block related signal pathways and prevent inflammation and reduce liver fibrosis (Li et al., 2018a). A recent observation proposed that APS can reduce PF through the TGF-β1/NF-kB pathway, which can effectively improve the deposition of COL-1, COL-3, and FN *in vivo*, APS reduce the area of fibrosis and HYP content in the matrix, and reduce the expression of a-SMA neutralizes E-cadherin, it significantly reduces the activation of the EMT and NF-kB pathways of TGF-β1 *in vitro* (Zhang et al., 2020). Therefore, the mechanism of APS protecting PF is the regulation of TGF-β1/NF-kB pathway.

### Polyporus Polysaccharide (PPS)

The pharmaceutical ingredients of polyporus umbellatus include polysaccharides, ergosterol, biotin, protein and other molecules, among which PPS is one of the main biologically active substances of polyporus umbellatus, the main components of PPS are (1–3)-β-Glc main chain and (1–6)-β-Glc side chain β-glucan, the Mw is about 1.6 × 10^5^ Da (Zong et al., 2012). Previous studies have reported various pharmacological activities of PPS, such as anti-tumor, anti-cancer, anti-oxidation and anti-inflammatory (Li et al., 2010; Guo et al., 2019). In renal fibrosis, PPS can improve the accumulation of TGF-β1, restore the balance of MMP/TIMP factors and improve renal fibrosis (Li et al., 2019a). PPS can significantly inhibit the ECM components in human lung fibrosis (HLFs) cells treated with TGF-β1, and inhibit Smad2/3 phosphorylation even reduce TβRI and TβRII, but it has no effect on TβRIV and can also inhibit medium MMP-2/MMP-9 expression (Jiang et al., 2020). Therefore, PPS prevented the further deterioration of PF through TGF-β1/Smad2/3/TβRI, and TβR II/MMP level signals.

### Rhodiolasachalinensis Polysaccharide A (RSA)

RSA is an acidic heteropolysaccharide isolated from rhodiola alpina, its mainly composed of Ara, Rha, Xyl, Glc, Gal, and GalA, the molar ratio is 1.00:3.23:0.26:0.34:0.84:10.24, and the relative Mw is about 50,000 Da (Han et al., 2002), has anti-oxidant, anti-viral, anti-oxidant and anti-tumor effects (Yang et al., 2016; Song et al., 2019). Previous evaluations have reported that salidroside protects mice from acute lung injury through the NF-kB pathway by controlling the production of inflammatory cytokines, and is very effective in preventing oxidative stress after exercise in mice (Guan et al., 2012). Studies have suggested that RSA can significantly inhibit the death rate of A549 cells induced by TGF-β1 and the expression of the mesenchymal marker Fibronection-EDA (Fn-EDA) (Li, et al., 2016). Rhodiola rosea significantly reduced the expression levels of MMP-9 and α-SMA in PF in a dose-dependent manner (Zhang et al., 2016). Therefore, RSA can improve the abnormality of Fn-EDA through TGF-β1 to achieve the purpose of treating PF.

### Polysaccharides From Dendrobium Officinale (PDO)

Dendrobium officinale is an epiphytic herb of the orchid family with mainly grows on semi-shaded moist rocks at an altitude of about 1,600 m in the Nanshan mountains of china. PDO is a neutral heteropolysaccharide isolated from the Chinese medicinal material dendrobium candidum, with composed of (1→4)-linked Man and (1→4)-linked Glc, the molar ratio is 5.9:1 and the Mw is 1.78 × 10^5^ Da (He et al., 2016; Yu W. et al., 2018). It has the effects of anti-oxidation, anti-tumor, anti-viral, anti-aging, lowering blood sugar and promoting hair growth (Wang J.-H. et al., 2017; Liu et al., 2019b), and is often valued in the field of dermatology. Dendrobium nobile polysaccharides can improve the antioxidant capacity of rats and reduce liver inflammation, so it has an antagonistic effect on liver fibrosis (Sibinska et al., 2017). Early studies on dendrobium candidum can treat myocardial fibrosis (Zhang et al., 2016b; Zeng et al., 2020). PDO can reduce the number of neutrophils in lung tissue induced by BLM, improve inflammation and fibrosis, enhance Smad2/3 and inhibit the expression of pSmad2/3 protein through TGFβ1, thereby inhibiting the type II rat alveolar epithelial cells (RLE-6TN, CRL-2300) induced by TGF-β1 fibronectin and COL-1 (Chen et al., 2018). Therefore, PDO treatment of PF lies in the regulation of TGF-β1/Smad2, Smad3/pSmad2/3.

### Bletilla Striata Polysaccharide (BSP)

Two water-soluble polysaccharides, BSP-1 and BSP-2, were extracted and purified from *bacillus* striata tuber, and their Mw are 8.354 × 10^4^ and 1.26 × 10^4^ Da, both BSP-1 and BSP-2 are composed of Man and Glc, and the molar ratio are 4.0:1.0 and 3.0:1.0. The backbone is mainly composed of repeated β-1,4-linked d-Man residues and β-1, 4-linked d-Glc residue composition. It has anti-ulcer, anti-oxidation, anti-inflammatory, anti-tumor and immunomodulatory activities (He et al., 2017; Liu et al., 2020). BSP can protect renal fibrosis through the down-regulation of TβRI, TβRII and a-SMA mediated by TGF-β (Wang et al., 2014). Some researchers have confirmed that a Mw of 2.23 × 10^5^ kDa, BSP can reduce cell recruitment to protect lung tissue inflammation, the content of HYP is significantly reduced, TGF-β3 and TNF-β1 are also improved, they also proved that bletilla striata extract also has a certain degree of resistance the role of fibrosis (Guo et al., 2016). Therefore, BSP improves lung inflammation and abnormal cell recruitment through TGF-β/TNF to improve lung tissue fibrosis.

### Seaweed Sulfated Oligosaccharide (MS80)

Marine macroalgae are mainly divided into four parts, green algae, cyanobacteria, brown algae and red algae. MS80 is a new type of sulfated oligosaccharide (1→4α-d-Glc) extracted from seaweed, with the Mw is 8,000 Da, MS80 can anti-cancer, anti-inflammatory and anti-tumor (Chen et al., 2020). MS80 inhibits BLM-induced PF *in vivo*, antagonizes TGF-β1-induced human embryo pulmonary fibroblast (HEPF) cells proliferation *in vitro*, prevents COL deposition and MMP activity, and inactivates ERK and p38 signaling pathways (Jiang and Guan, 2009). In addition, the MS80 targeting protein is receptor interacting protein 2 (a key component of CD40 signal transduction). MS80 inhibits the activation of NF-kB induced by CD40 linkage, thereby inhibiting the secretion of inflammatory cytokines, COL synthesis and fibrogenesis the excessive proliferatsion of cells confirms that MS80 has anti-fibrosis effects both *in vivo* and *in vitro* (Du et al., 2010). MS80 can also effectively inhibit standardized TGF-β1/Smad signaling, thereby improving the changes in EMT marker levels (Zhou et al., 2016). Therefore, MS80 can improve PF through TGF-β1/MMP/ERK/NF-kB/p38.

### Low Molecular Weight Fucoidan (LMWF)

LMWF is a sulfated polysaccharide extracted from brown algae, the Mw is 8–10 kDa, containing Rha, Fuc, Xly, Man, Glc, Gal, GlcA and Gala, the linkage is backbone →3)-Galp-(1→, →6)-Glcp-(1→, →6)-Galp-(1→, →3,6)-manp-(1→ with →3)-Fucp-(1→, →4)-Glcp-(1→ and sulfated end units (Chen et al., 2020), consists of (1→3)-linked α-thiopropionyl residues or alternating (13→)- and (→14)-linked α-thiophosphoryl residues. Depending on previous studies, LMWF can lower blood sugar, anti-inflammatory, and promote angiogenesis (Haddad et al., 2015; Wang et al., 2018; Zheng et al., 2018). LMWF has an antagonistic effect on liver fibrosis through the TGF-β1/Smad3 pathway (Hayashi et al., 2008). Its also lnhibit the proliferatsion of breast cancer cells and the expression of EMT biomarkers (Hsu et al., 2013). In addition, LMWF can reduce inflammation of the inflammatory cytokines TIMP-1, chemokine ligand 1 (CXCL1), monocyte chemotactic protein-1 (MCP-1), and macrophage inflammation protein-2 (MIP-2) of lung tissue, TNF content to improve radiation-induced pneumonia in mice and further PF (Yu H.-H. et al., 2018). LMWF inhibits the morphological changes and proliferatsion of A549 cells induced by TGF-β1. In the male C57BL/6 mice model, LMWF attenuates the lung EMT phenotype. LMWF down-regulates TGF-β1/ERK signals *in vivo* and *in vitro* to regulate PF (Wang et al., 2019a). Therefore, LMWF treats PF through TGF-β1/ERK signal and improves inflammatory cytokines in pathological lung tissue.

### Angelica Sinensis Polysaccharide (ASP)

ASP is an acidic heteropolysaccharide extracted from the root of angelica sinensis, its composed of GalA, Glc, Ara and Gal with the molar ratio of 1.00:1.70:1.85:5.02, the Mw is 80 kDa. Its main chain is composed of (1→3)-connected Galp, (1→6)-connected Galp and 2-OMe-(1→6)-connected Galp, with three branches connected to 2-OMe-(- 1→6) link Galp and terminate with GlcpA and Araf (Zhang et al., 2016a). ASP has immune regulation, anti-tumor, anti-oxidation and anti-proliferatsion functions (Li M. M. et al., 2020; Xu et al., 2021). There is also the ability of the compound Danggui Buxue Decoction total glycosides (containing astragalus and angelica) to inhibit abnormal lung tissue ECM and reverse the expression of MMP/TIMP (Gao et al., 2012). ASP can improve the content of hyaluronic acid and laminin in the lung tissue of rats with PF induced by BLM (Wang et al., 2010). The latest report points out that ASP inhibits PF by down-regulating the expression of differentiation-antagonizing non-protein coding RNA (DANCR), which inactivates FOXO3 translation after transcription in an AU binding factor 1 (AUF1)-dependent manner (Qian et al., 2020). Therefore, ASP can inhibit EMT and PF through the TGF-β1/DANCR/AUF-1/FOXO3 regulatory axis.

### Total Polysaccharide From O, Lanpingensis (POL)

POL is an insect fungal polysaccharide isolated from cordyceps Lanping. It is mainly distributed in northwestern Yunnan, china as a new species reported in recent years, which is closely a relative species of cordyceps sinensis. POL Mw is 3.2 × 10^5^ Da, composed of Gal, Man and Glc, and the molar ratio is 5.30:13.38:81.31 (Zhou et al., 2020). It was reported earlier that POL has an anti-inflammatory effect, which can improve liver fibrosis by alleviating the body’s oxidative stress, reduce inflammation and anti-apoptosis of liver cells (Zhang et al., 2017). POL can Treat renal insufficiency by enhancing antioxidant capacity and improving immune regulation ability (Ying-Mei et al., 2020). Ophiocordyceps lanpingensis can treat respiratory diseases. POL can significantly reduce the content of collagen in lung tissue and inflammation of lung tissue by inhibiting classically activated macrophages 1 (M1) and alternately activated M2 in lung tissue The recruitment of PF inhibits the occurrence of PF (Zhou et al., 2020). Therefore, POL can improve lung inflammation by inhibiting the excessive recruitment of M1 and M2 under TGF-β1 to achieve a therapeutic effect.

### Lycium Barbarum Polysaccharide (LBP)

In the wolfberry extract, LBP isolated from the fruit of lycium barbarum caused the biological activity of the wolfberry. LBP is a group of water-soluble sugar conjugates with a Mw is 10–2,300 kDa, accounting for 5–8% of dried fruit. LBP glycan backbone is mainly represented by α-(1→4)-galA, α-(1→6)-glc, β-(1→3)-galp (typical arabinogalactan protein) and β-(1→6)-giap, other structures that are less representative are α-(1→5)-ara and β-(1→4)-galp, which have different branching and terminal sites (Tian et al., 2019). LBP has the effects of lowering blood sugar, lowering blood lipid, anti-oxidation and anti-tumor effect (Masci et al., 2018; Huang et al., 2019). LBP possibly inhibit the expression of genes such as COL-1 and α-SMA in inflamed lung tissues, weaken the excessive proliferatsion of fibroblasts and prevent their differentiation into myofibroblasts, thereby reducing the content of HYP in lung tissues to inhibit the development of PF in C57BL/6 mice (Liu et al., 2016b). Therefore, LBP inhibits PF by improving the degree of fibers in the lung tissue.

### Morel Polysaccharide-1 (FMP-1)

Morels belong to the category of mushrooms, with high nutritional value and delicious taste, both the flavor and the pharmacological effects have been highly regarded (Tietel and Masaphy, 2018). FMP-1 is a heteropolysaccharide in morchella fruiting body, the Mw is 4.7 × 10^3^ Da, composed of Man, Glc, and Gal with the molar ratio of 1.00:7.84:1.24, the main chain is 1,4-linked Glcp and 1,6-connected Galp composition (Cai et al., 2018). Previous studies have shown that morel contains many biologically active ingredients, such as polysaccharides, protein, dietary fiber and vitamins. FMP-1 has immunomodulatory, anti-oxidant, anti-value-added and anti-tumor effects (C. Liu et al., 2016a; Li et al., 2017). Controlling oxidative stress after lung injury has been shown to be effective in inhibiting fibrosis, it is proposed that polysaccharides can activate antioxidant defenses and improve oxidative stress damage (Hao et al., 2016; Chen et al., 2017). The phosphatidylinositol 3-kinase (PI3K)/AKT pathway is one of the effective ways to fight oxidative stress (Mozaffari et al., 2010), and it has a protective effect on lung epithelial cell death induced by oxidative stress (Deng et al., 2013b). Many examines have confirmed that the up-regulation of heme oxidase-1 (HO-1) is involved in the cells defense mechanism against the results of oxidation (Lee et al., 2012; Jang et al., 2016). Related1 research reports that FMP-1 can promote nuclear factor erythroid 2-related factor 2 (Nrf2) phosphorylation and nuclear translocation, and up-regulate downstream protein HO-1 through the PI3K/AKT-Nrf2 signaling pathway, thereby protecting human alveolar epithelial cells (A549) from hydrogen peroxide oxidative stress (Li et al., 2018c). Therefore, FMP-1 can be used as a natural potential antioxidant to treat PF through the Nrf2/HO-1/PI3K/AKT signaling pathway.

### Ganoderma Lucidum Polysaccharide (GIP)

Ganoderma is an edible medicinal mushroom, which has been praised for 2000 years. It can enhance human vitality. GIP is a neutral heteropolysaccharide isolated from ganoderma lucidum. It is composed of Gal, Rha and Glc with the molar ratio of 1.00:1.15:3.22, the Mw is 78 kDa, and the backbone structure is mainly composed of 1,2-linked bL- Rhap, 1,3,6-linked aD-Galp, 1,2,6-linked aD-Glcp, and 1-link aD-Glcpconsisted mainly of 1,2-linked-β-L-Rhap, 1,3,6-linked-a-D-Galp, 1,2,6-linked-a-D-Glcp, and1-linked a-D-Glcp. (Pan et al., 2012). It is reported that a pure Glc polymer named β-glucan is considered to be one of the active ingredients of GIP (Zeng et al., 2019). GIP has many therapeutic effects on human diseases, including anti-oxidation, anti-inflammatory, anti-tumor, lowering blood sugar, lowering blood lipids, and anti-aging (Wang et al., 2017b; Sohretoglu and Huang, 2018; Zeng et al., 2018). In addition, it has been reported in the literatsure that GIP 100–300 mg/kg can reverse PF after 28 days. The main mechanism is related to the increase of lung antioxidant capacity. GIP can increase pathological lung tissue CAT (catalase), SOD (superoxide dismutation), GSH (glutathione), and GSH-Px (glutathione peroxidase) levels, simultaneously, reduce the content of MDA (malondialdehyde) and HYP in lung tissue (Chen et al., 2016). Later reports confirmed that ganoderma lucidum has obvious protective effect on oxidative damage caused by oxidants (Laçin et al., 2019; Lin and Deng, 2019). Ganoderic acid can treat renal fibrosis through TGF-β/Smad and MAPK signaling (Geng et al., 2020). Therefore, GIP improves the status of PF by improving the lung’s antioxidant capacity.

### Lily Polysaccharide (LLP)

LLP-1, LLP-2 and LLP-3 are the three new polysaccharide components in lily, their Mw are estimated to be 2.25 × 10^6^, 2.02 × 10^6^, and 2.08 × 10^6^ Da, LLP-1 and LLP-2 are mainly composed of Glc and Man, with the molar ratio of 1:2 and 1:1, respectively, LLP-3 is mainly composed of Ara, Gal, Glc, and Man, with the molar ratio is 2:2:2:1 (Chen et al., 2014; Hou et al., 2016). LLP has a variety of activities, such as hypoglycemic, anti-oxidant and anti-cancer effects (Li et al., 2020b). LLP can improve the PF alveolar compartment and reduce the infiltratsion of inflammatory cells. The mechanism is to inhibit the protein expression of MMP-9 and TIMP-2 in the fibrosis model (Luo, et al., 2013a). LLP combined with bone marrow mesenchymal stem cell transplantation inhibits the expression of TNF-α and NF-kB in the lung tissue of PF mice, improves the recruitment of COL and reduces the content of HYP. LLP can reduce the pathological damage of PF (Luo, et al., 2013b). Therefore, LLP treats PF by adjusting the balance of MMP/TIMP in rats, improving the NF-kB signaling pathway and inflammatory TNF-a abnormalities in lung tissue.

### Yupingfeng–Polysaccharide (YPF–p)

Yupingfeng powder is a well-known TCM compound consisting of *Astragalus*, Atractylodes and Fangfeng. There are many compounds in Yupingfeng powder, including total polysaccharides, total saponins and volatile oil. Among them, YPF-p is one of the important components extracted from Yupingfeng powder, it has anti-inflammatory, anti-allergic, immune-regulating and alleviating effects of lung qi deficiency (Sun et al., 2016; Fan et al., 2017). Anti-fibrosis studies have found that YPF-p can improve the level of HYP in Wistar rats induced by pingyangmycin hydrochloride and the content of COL-3, COL-4, laminin, and hyaluronic acid in serum, and can down-regulate Wistar rats lungs. TGF-β1 mRNA expression in tissues (Wang et al., 2009). The mechanism presumably is that inhibit the increase of TGF-β1 mediated fibroblast activation and thus reduce synthesis (Xu et al., 2014). In addition, the total glycosides of Yupingfeng reduced the protein expression of box1 in the high mobility group, and reversed TGF-β1 to improve PF (Cui et al., 2015; Li et al., 2015). Therefore, YPF-p treats PF by down-regulating the expression of TGF-β1 mRNA and improving the excessive aggregation of fibroblasts induced by TGF-β1.

## Conclusion and Future Prospects

These polysaccharides can significantly improve the abnormal recruitment and apoptosis of various cells in the lung tissue induced by the TGF-β signaling pathway, regulate the imbalance of the body caused by lung inflammation, and can control lung tissue damage through oxidative stress, thus confirming these polysaccharides can stabilize PF lung function and prevent further damage.

Our understanding of this evolving deadly disease is constantly improving, but effective treatments are still elusive. PF is also one of the main complications of COVID-19. Studying the properties of polysaccharides and combining polysaccharides with other drugs may provide medical help for PF patients with COVID-19. The epidemiological risk factors and biological process of PF and COVID-19 are similar. After severe acute respiratory syndrome coronavirus 2 (SARS-CoV-2) induced pneumonia may cause idiopathic PF related to its physical damage. Hence, modulating the mechanism of fibrosis in SARS-CoV-2 infection to exert a therapeutic effect may be acceptable. The chemical structure of Chinese herbal medicine polysaccharides is the basis of biological activity. Using the hydroxyl, carboxyl, amino, and other groups of sugar residues to modify the structure of polysaccharide molecules on the surface of polysaccharide molecules, For example, the GIP isolated from Fudan-Yueyang-G. lucidu has undergone methylation analysis, periodate oxidation, Smith degradation, and NMR characterization analysis to obtain a neutral polysaccharide with four residues A, B, C, and D which is significantly enhanced improve the lung’s antioxidant capacity to achieve the purpose of treating PF, The structure determines the activity, can makes the multiple structure of the plant clearer, and seeks the regularity between the nanostructure and the biological activity to improve the immune activity of polysaccharides and reduce toxic effects. Chemical polysaccharides with different structures have great differences in biological activity. Further explore the relationship between the biological effects and efficacy of Chinese herbal medicine polysaccharides to explain the therapeutic mechanism of polysaccharides in PF. It is very important to provide a certain reference for the in-depth study and exploration of the structure-activity relationship of Chinese herbal medicine polysaccharides and the development and application of carbohydrate products. This provides important medical theory and economic value for the development of Chinese herbal polysaccharides to treat fibrotic diseases, and has a very important society. Polysaccharides can be a new type of anti-fibrosis treatment drugs. While, the structure of traditional Chinese medicine polysaccharides is complex and diverse, and its special active mechanism needs to be further studied. The establishment of specific, efficient and practical methods for the stability of polysaccharides is of great significance to the research and development of modern medicine.

## Glossary

**PF**: Pulmonary fibrosis
**TCM**: Traditional Chinese medicine
**HYP**: Hydroxyproline
**ECM**: Extracellular matrix
**TGF-β**: Transforming growth factor-β
**BLM**: Bleomycin
**COL**: Collagen
**EMT**: Epithelial-mesenchymal transition
**a-SMA**: a-smooth muscle actin
**GPS**: Ginseng polysaccharide
**BPS**: Basil polysaccharide
**APS**: *Astragalus* polysaccharide
**PPS**: Polyporus polysaccharide
**RSA**: Rhodiolasachalinensis polysaccharide A
**PDO**: Polysaccharides from dendrobium officinale
**BSP**: Bletilla striata polysaccharide
**MS80**: Seaweed sulfated oligosaccharide
**LMWF**: Low molecular weight fucoidan
**ASP**: Angelica sinensis polysaccharide
**POL**: Total polysaccharide from O, lanpingensis
**LBP**: Lycium barbarum polysaccharide
**FMP-1**: Morel polysaccharide-1
**GIP**: Ganoderma lucidum polysaccharide
**LLP**: Lily polysaccharide
**YPF-p**: Yupingfeng-polysaccharide
**TβR1**: Transforming growth factor-β1 receptor
**HLFs**: Human lung fibrosis
**HEPF**: Human embryo pulmonary fibroblast
**DANCR**: Differentiation antagonistic non-protein coding RNA
**Mw**: Molecular weight
**Nrf2**: Nuclear factor erythroid 2-related factor 2
**AUF1**: AU binding factor 1
**MMP**: Matrix metalloproteinase
**TIMP**
Tissue inhibitor of metalloproteinase
**NF-kB**: Nuclear factor-kB
**IL**: Interleukin
**MCP-1**: Monocyte chemoattractant protein-1
**MDA**: Malondialdehyde
**ROS**: Reactive oxygen species
**SOD**: Superoxide dismutase
**TNF**: Tumor necrosis factor
**Ho-1**: Heme oxygenase-1
**AKT**: Protein kinase B
**ERK**: Extracellular signal-regulated kinase
**FN**: Fibronectin
**MIP-2**: Macrophage inflammatory protein-2
**M1**: Macrophages 1
**CXCL1**: Chemokine ligand 1
**PI3K**: Phosphatidylinositol 3-kinase
**CAT**: Catalase
**GSH**: Glutathione
**GSH-Px**: Glutathione peroxidase
**MAPK**: Mitogen -activated protein kinase
**AP-1**: Activator protein-1
**Ara**: Arabinose
**Glc**: Glucose
**Rha**: Rhamnose
**Man**: Mannose
**Gal**: Galactose
**Xyl**: Xylose
**GalA**: Galacturonic acid
**Fru**: Fructose
